# Novel Peripherally Derived Neural‐Like Stem Cells as Therapeutic Carriers for Treating Glioblastomas

**DOI:** 10.5966/sctm.2016-0007

**Published:** 2016-09-14

**Authors:** Alexander Birbrair, Anirudh Sattiraju, Dongqin Zhu, Gilberto Zulato, Izadora Batista, Van T. Nguyen, Maria Laura Messi, Kiran Kumar Solingapuram Sai, Frank C. Marini, Osvaldo Delbono, Akiva Mintz

**Affiliations:** ^1^ Ruth L. and David S. Gottesman Institute for Stem Cell and Regenerative Medicine, Albert Einstein College of Medicine, Bronx, New York, USA; ^2^ Department of Cell Biology, Albert Einstein College of Medicine, Bronx, New York, USA; ^3^ Department of Internal Medicine‐Gerontology, Wake Forest School of Medicine, Winston‐Salem, North Carolina, USA; ^4^ Department of Pathology, Federal University of Minas Gerais, Minas Gerais, Brazil; ^5^ Department of Radiology, Wake Forest School of Medicine, Winston‐Salem, North Carolina, USA; ^6^ Brain Tumor Center of Excellence, Comprehensive Cancer Center of Wake Forest University, Winston‐Salem, North Carolina, USA; ^7^ Department of Cancer Biology, Wake Forest School of Medicine, Winston‐Salem, North Carolina, USA; ^8^ Wake Forest Institute for Regenerative Medicine, Winston‐Salem, North Carolina, USA

**Keywords:** Glioblastoma, Cellular therapy, Muscle stem cells, Neural differentiation, Neural‐like stem cells

## Abstract

Glioblastoma (GBM), an aggressive grade IV astrocytoma, is the most common primary malignant adult brain tumor characterized by extensive invasiveness, heterogeneity, and angiogenesis. Standard treatment options such as radiation and chemotherapy have proven to be only marginally effective in treating GBM because of its invasive nature. Therefore, extensive efforts have been put forth to develop tumor‐tropic stem cells as viable therapeutic vehicles with potential to treat even the most invasive tumor cells that are harbored within areas of normal brain. To this end, we discovered a newly described NG2‐expressing cell that we isolated from a distinct pericyte subtype found abundantly in cultures derived from peripheral muscle. In this work, we show the translational significance of these peripherally derived neural‐like stem cells (NLSC) and their potential to migrate toward tumors and act as therapeutic carriers. We demonstrate that these NLSCs exhibit in vitro and in vivo GBM tropism. Furthermore, NLSCs did not promote angiogenesis or transform into tumor‐associated stromal cells, which are concerns raised when using other common stem cells, such as mesenchymal stem cells and induced neural stem cells, as therapeutic carriers. We also demonstrate the potential of NLSCs to express a prototype therapeutic, tumor necrosis factor α‐related apoptosis‐inducing ligand and kill GBM cells in vitro. These data demonstrate the therapeutic potential of our newly characterized NLSC against GBM. Stem Cells Translational Medicine *2017;6:471–481*

**Significance Statement**

Radiation and chemotherapy have failed to cause significant therapeutic benefit in glioblastoma patients because of their limitations. This has spurred investigations into the use of engineered stem cells to deliver antitumor drugs as a complementary strategy. This study reports that neural‐like stem cells isolated from skeletal muscles show tumor‐tropism and can be engineered to secrete therapeutics but do not form tumors and do not transform into tumor‐ associated cells or cause angiogenesis, in contrast to commonly used stem cells. These advantages coupled with the therapeutic potential of neural‐like stem cells highlights their ability to be used as therapeutic carriers against glioblastomas.

## Introduction

Glioblastoma (GBM) is an aggressive primary malignant astrocytoma characterized by infiltrating margins and angiogenesis. GBMs are a heterogeneous mixture of tumor and normal cells including activated macrophages and fibroblasts 1, 2. GBM often recurs because of invasive tumor cells and micrometastasis, which persist upon treatment and subsequently form aggressive tumors that are resistant to standard therapy. Standard treatment options such as surgical resection and wide local radiotherapy only marginally improve patient survival, but the exposure of healthy brain tissue to radiation causes a decline in cognition as well as other severe neurological complications 3.

Although GBM is often characterized by extensive angiogenesis, systemic delivery of chemotherapeutics has been disappointing because of the protective nature of the blood‐brain barrier and highly tortuous structure of newly formed tumor blood vessels 4. Because of the ineffectiveness of existing treatment options, extensive research is being carried out in the use of targeted cytotoxins, small molecules, redirected T cells, and stem cell‐based therapies against GBM 5, 6.

Studies suggest the role of stem and progenitor cells in the initiation and maintenance of various tumor types including GBM 7. Migration of stem cells to the tumor microenvironment has been attributed to their affinity to tumor‐secreted cytokines and intratumoral hypoxic regions 8. Neural stem cells (NSCs) showed therapeutic potential due to their GBM‐homing properties when transplanted locoregionally in various studies, yet the implausibility of isolating autologous NSCs from GBM patients severely limits their translational potential 9. Therefore, peripherally derived stem cells and genetically reprogrammed cells are being commonly used for stem cell therapies. Genetically engineering GBM‐tropic stem cells would allow for systemic or locoregional delivery of therapeutics to various tumors, and such alternate treatment strategies are currently undergoing clinical testing after showing promise in preclinical studies.

Mesenchymal stem cells (MSCs) are the most commonly used peripherally derived stem cells as therapeutic carriers and show great advantage in their ease of isolation and culture in vitro 10. However, potential drawbacks of using MSCs as therapeutic carriers are that naïve and genetically engineered MSCs promote angiogenesis and tumor progression by transforming into tumor‐associated fibroblasts (TAFs) 11, 12. In addition to peripherally derived stem cells, induced pluripotent stem cells and induced neural stem cells have also shown promise in their migratory capability toward tumors in preclinical studies, yet there is concern owing to the possibility that they can form tumors or aid established tumor growth upon being exposed to tumor exosomes, morphogens, and cytokines within the tumor microenvironment.

In this study, we examined the GBM tropism of newly isolated neural‐like stem cells (NLSC), recently discovered by our group in skeletal muscle cultures of transgenic mice using orthotropic GBM mouse models. Our results also show that NLSCs do not promote angiogenesis or transform into TAFs. We further showed the ability to genetically engineer NLSCs to secrete a prototype therapeutic and thus highlight their potential to deliver antitumor therapeutics. NLSCs may therefore show a significant advantage over other commonly used stem cells for their use as carriers of anti‐GBM therapeutics.

## Materials and Methods

### Animals

Our colony of Nestin‐green fluorescent protein (GFP) transgenic mice were maintained homozygous for the transgene on the C57BL/6 genetic background (Grigori Enikolopov, Cold Spring Harbor Laboratory, Cold Spring Harbor, NY, https://www.cshl.edu). For transplantation experiments, male athymic nude mice were purchased from Taconic Farms (Hudson, NY, http://www.taconic.com). All colonies were housed in a pathogen‐free facility of the Animal Research Program at Wake Forest School of Medicine under a 12:12‐hour light/dark cycle and fed ad libitum.

### Isolation and Dissociation of Flexor Digitorum Brevis Muscle

Flexor digitorum brevis muscles from Nestin‐GFP transgenic C57BL/6 mice were excised and used to culture Nestin‐GFP^+^NLSCs. Flexor digitorum brevis muscles were dissected after carefully separating them from their surrounding connective tissue. The muscles were then minced and digested by gentle agitation in 0.2% (wt/vol) type 2 collagenase (Worthington Biochemical, Lakewood, NJ, http://www.worthington‐biochem.com) diluted in Krebs solution at 37°C for 2 hours. Later, they were resuspended in growth medium and dissociated by gentle trituration.

### Isolation of Homogenous Population of GFP^+^ NLSCs From Transgenic Nestin‐GFP^+^ C57BL/6 Mice Using Fluorescence‐Activated Cell Sorting

Fluorescence‐activated cell sorting (FACS) was performed 14 days after flexor digitorum brevis muscle dissociation to isolate Nestin‐GFP^+^ NLSCs. Cultured flexor digitorum brevis muscle‐derived cells were washed with phosphate‐buffered saline (PBS) and treated with 0.25% trypsin/0.05% EDTA (Thermo Fisher Scientific Life Sciences, Waltham, MA, http://www.thermofisher.com) to isolate them in a suspension. We applied mechanical trituration using fire‐polished glass pipettes to increase cell dissociation. Cells were centrifuged at 1,000 rpm for 5 minutes, and the pellet was resuspended in Dulbecco's modified Eagle's medium (DMEM; Thermo Fisher). Aggregates were removed by passing them through a 40‐μm cell strainer (BD Biosciences, East Rutherford, NJ, https://www.bdbiosciences.com) prior to sorting. FACS was carried out on a FACS Aria Sorter (BD Biosciences) at 4°C and a pressure of 20 ψ, using a laser at the 488‐nm line, a 530/30 band‐pass filter, a 100‐μm sorting tip, and a 34.2‐kHz drive frequency, sterilized with 10% bleach. Data acquisition and analyses were performed by using BD FACS Diva 5.0.3 software, gated for a high level of GFP expression. Cells were sorted twice to attain a highly pure GFP^+^ population and were analyzed after sorting to confirm that all were GFP^+^.

### Homogenous Population of NLSCs Can Be Isolated From Skeletal Muscle of Transgenic Nestin‐GFP^+^C57BL/6 Mice Using Antinerve Growth Factor Receptor (p75) Antibody‐Based FACS

Nestin‐GFP^+^ NLSCs were isolated as mentioned earlier and cultured for 14 days in laminin‐coated dishes containing growth media. Cells were removed from the flask using Versene (Thermo Fisher), counted, spun down at 5,000 rpm for 5 minutes in microfuge tubes, and resuspended in 100 µl 1% PBS‐bovine serum albumin (BSA) per 2 × 10^5^ cells. Aggregates were removed by passing them through a 40‐μm cell strainer prior to sorting. Cells were then incubated on ice for 1 hour and later labeled with 5 μg/ml of rabbit antinerve growth factor receptor (anti‐NGFR) antibody (Advanced Targeting Systems, San Diego, CA, http://atsbio.com) for 2 hours. After three washings with 1% PBS‐BSA, cells were labeled with Alexa Fluor 647 anti‐rabbit antibody (Thermo Fisher) for 1 hour in the dark on ice. After three washings with 1% PBS‐BSA, cells were resuspended in 1% PBS‐BSA and sorted using a FACS Aria Sorter. Data acquisition and analyses were performed using BD FACS Diva 5.0.3 software.

### In Vitro Migration Assay

NLSCs were plated on the upper layer of cell‐permeable fluorescent filters of a 24‐well transwell plate coated with Matrigel (BD Biosciences). Chambers below the filters were filled with conditioned media from G26H2 murine glioblastoma cell line and serum‐free media. After an incubation period of 24 hours at 37°C, chemotaxis of NLSCs toward GBM cell line conditioned media was evaluated by counting the number of GFP^+^ cells on the plate surface using a fluorescence microscope.

### In Vivo Migration of GFP^+^ NLSCs Toward DsRed^+^ U87 Human Glioblastoma

Orthotropic glioblastomas were implanted by stereotaxic implantation of 2 × 10^5^ actively growing DsRed^+^ U87 glioma cells in nude mice. Briefly, mice were weighed and anesthetized with a mixture of 114 mg/kg ketamine and 17 mg/kg xylazine. Mice were placed in a stereotaxic setup, and a hole was made 2 mm lateral and 0.5 mm posterior to the bregma in the right cerebral hemisphere through a scalp incision. Stereotaxic injection was performed using a Just for Mice stereotaxic apparatus (Harvard Apparatus, Holliston, MA, http://www.harvardapparatus.com), with injection using a 10‐µl syringe (Hamilton, Reno, NV, http://www.hamiltoncompany.com) through the hole to a depth of 3.2 mm. A Nanomite programmable syringe pump (Harvard Apparatus) delivered constant infusion at a rate of 0.5 µl/min to a total volume of 5 µl. All mice received an analgesic (ketaprofen) and were monitored for body weight and ambulatory, feeding, and grooming activities. Seven days’ posttumor implantation, 5 × 10^5^ Nestin‐GFP^+^ NLSCs were implanted 2 mm apart from the tumor location using the same stereotactic apparatus and injection rate as the cell implantations. Mice were monitored for loss of weight and were euthanized after they had lost 25% of body weight according to institutional animal care and use committee guidelines. Brains for these mice were extracted, sectioned, and observed under a fluorescence microscope to visualize migration of implanted GFP^+^ NLSCs toward established orthotopic DsRed^+^ tumors.

### Formation of Endothelial Tubular Network In Vitro

Endothelial tubular network formation assay to compare angiogenic potential of NLSCs to MSCs was performed, as has been described earlier 13. After overnight incubation at 4°C, 150 µl of growth factor‐reduced Matrigel was transferred to a 48‐well plate on ice. After incubating the plate at 37°C for 30 minutes to allow gel formation, 4 × 10^5^ human umbilical vein endothelial cells (HUVECs) were diluted in cell culture medium, and 500 µl of the cell suspension was transferred to each well of the 48‐well plate. HUVECs were cocultured with 1.5 × 10^5^ purified GFP^+^ human MSCs and 1.5 × 10^5^ purified NLSCs. HUVECs cultured alone were used as a control, and the plate was incubated at 37°C for 3 hours. After incubation, culture medium was gently removed, and 150 µl of additional Matrigel was added to form a sandwich, which was finally covered with 500 µl of culture medium. After 10 days, formation of endothelial tube networks was observed under a microscope.

### Matrigel Plug Assay

Matrigel plug assay was performed, as has been described earlier 14. We suspended 4 × 10^6^ HUVECs with purified GFP^+^ human MSCs and NLSCs (5 × 10^5^) in 500 µl of cell culture medium. The cell suspensions were mixed with 500 µl of precooled growth factor‐reduced Matrigel at a ratio of 1:1 at 4°C. One milliliter of this mixture was injected subcutaneously into the dorsal region of 2‐ to 3‐month‐old nude mice to generate Matrigel plugs. After 2 weeks, plugs were recovered; blood vessel formation was compared by observing them under a microscope.

### Fibrogenic Induction In Vitro

Dishes were coated with 1 ml of precooled laminin (0.02 mg/ml) and incubated at 37°C for 3 hours. After incubation, remaining laminin was removed. Two milliliters of differentiation medium (DMEM‐high glucose [Corning, Manassas, VA, https://www.corning.com], supplemented with 2% horse serum, 1% penicillin‐streptomycin [Pen‐Strep] [both Thermo Fisher], and 2.5 ng/ml transforming growth factor [TGF]‐β1 [Sigma‐Aldrich, St. Louis, MO, https://www.sigmaaldrich.com]) was added to each dish. We transferred 2.25 × 10^4^ human MSCs and 2.25 × 10^4^ NLSCs to separate dishes.

### Immunocytochemistry

After incubating the dishes at 37°C for 10 days, cells were fixed in 4% paraformaldehyde (PFA) for 30 minutes, permeabilized in 0.5% Triton X‐100 (Sigma‐Aldrich), and blocked using 5% (v/v) goat serum‐PBS overnight (Jackson Laboratories, Bar Harbor, ME, https://www.jax.org) at 4°C. Next day, cells were incubated with α‐smooth muscle actin (SMA) (Abcam, Cambridge, U.K., http://www.abcam.com) and collagen type 1 (Bio‐Rad, Raleigh, NC, https://www.bio‐rad‐antibodies.com) primary antibodies at room temperature for 4 hours and visualized by incubating for 2 hours with species‐specific secondary antibody conjugated with Alexa Fluor 568 (Thermo Fisher) at 1:1,000 at room temperature in the dark. Dishes were counterstained with Hoechst 33342 at 1:2,000 dilution (Thermo Fisher) and examined under a fluorescent microscope.

### Tumor Necrosis Factor α‐Related Apoptosis‐Inducing Ligand mCherry Secretion

In order to demonstrate the capability of NLSCs to be engineered for therapeutic purposes, we transfected NLSCs with a plasmid to induce expression of tumor necrosis factor α‐related apoptosis‐inducing ligand (TRAIL)‐mCherry. NLSCs were cocultured with U251‐Luc GBM cell line in a six‐well plate, and the cytotoxic effect due to the successful expression of TRAIL‐mCherry by NLSCs was examined by counting the number of viable Luc^+^ GBM cells and total luciferin signal in each well after 24 hours.

### Microscopy

An inverted motorized fluorescent microscope (Olympus IX81; Olympus, Tokyo, Japan, http://www.olympus‐global.com) with an Orca‐R2 Hamamatsu CCD camera (Hamamatsu Phototonics, Hamamatsu, Japan) and a laser scanning confocal microscope (Olympus FluoView1200; Olympus) were used for image acquisition. Camera drive and acquisition were performed using a MetaMorph Imaging System (Olympus), and Fluo View Viewer 4.2 software was used for image acquisition.

### Cell Culture

NLSCs were grown on laminin‐coated dishes in DMEM‐high glucose, supplemented with 2% l‐glutamine, 1% Pen‐Strep, 10% (vol/vol) horse serum (all Thermo Fisher), and 0.5% (vol/vol) CEE (Gemini Bio‐Products, West Sacramento, CA, http://www.gembio.com). G26‐H2 murine glioblastoma cell line, GFP^+^ human mesenchymal stem cells, and DsRed^+^ U87 cells were cultured in DMEM‐high glucose media, supplemented with 10% fetal bovine serum (Thermo Fisher) and 1% Pen‐Strep (Thermo Fisher). HUVECs were cultured in Medium 200 (Thermo Fisher).

### Cryopreservation

Mice were anesthetized, transcardially perfused with PBS, and followed by 4% PFA. Brains were removed, postfixed in 4% PFA, cryoprotected in 30% sucrose, covered with tissue embedding medium, snap frozen in liquid nitrogen, and stored at −80°C.

### Statistical Analysis

Results were expressed as mean ± SEM, and statistical significance was assessed using Student's *t* test with GraphPad Prism. We considered *p* < .05 as significant, and *p* < .01 as highly significant.

## Results

### Homogenous Population of NLSCs Can Be Isolated From Skeletal Muscles of Transgenic Nestin‐GFP^+^ C57BL/6 Mice Using Endogenous GFP‐Based FACS

**Figure 1:**
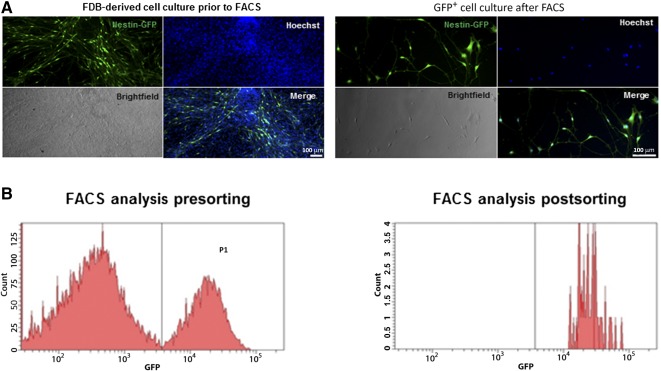
Isolation of Nestin‐GFP^+^ neural‐like stem cells (NLSCs) from FDB muscle cultures derived from Nestin‐GFP transgenic mice. **(A):** Unsorted FDB‐derived cell culture is shown in the left panel. NLSCs are green; all cells are counterstained with Hoechst nuclear stain (blue color). The merged picture (left panel) shows the abundance of Nestin‐GFP^+^ cells in the heterogeneous cell mixture derived from FDB muscles. Homogenous population of Nestin‐GFP^+^ NLSCs post FACS is shown in the right panel. NLSCs are green and counterstained with Hoechst nuclear stain (blue color). Scale bars = 100 μm. **(B):** Representative histograms for FDB culture before and after FACS. Histograms show the purity of the obtained GFP^+^ cell population. Abbreviations: FACS, fluorescence‐activated cell sorting; FDB, flexor digitorum brevis; GFP, green fluorescent protein.

We initially isolated NLSCs, which were the only GFP^+^ cells in cultures of skeletal muscles derived from transgenic Nestin‐GFP^+^ mice using FACS 15, 16, 17. Flexor digitorum brevis (FDB) muscle cell cultures derived from transgenic Nestin‐GFP^+^ mice were allowed to grow for 14 days, after which we performed FACS to isolate GFP‐expressing NLSCs. Initially, the skeletal muscle cell population was heterogeneous with a mixture of GFP^+^ and GPF^−^ cells (Fig. 1A). Following GFP expression‐based FACS, a pure cell population of Nestin‐GFP^+^ NLSCs was obtained (Fig. 1A) and extensively characterized in our prior work 15. The FACS histograms illustrate the purity of the isolated NLSC population and affirm that our results in subsequent experiments are attributed exclusively to the properties of peripherally derived NLSCs (Fig. 1B). After double FACS, GFP^+^ cells were reanalyzed under a fluorescence microscope and used for further experiments.

### Homogenous Population of NLSCs Can Be Isolated From Skeletal Muscle Using Anti‐ NGFR (p75) Antibody‐Based FACS

**Figure 2:**
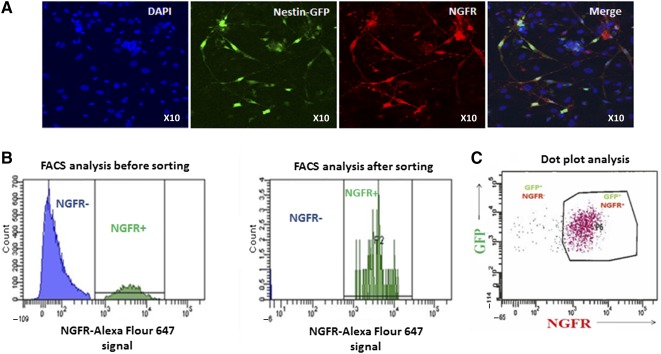
Isolation of GFP^+^ neural‐like stem cells (NLSCs) from Nestin transgenic mice using NGFR (p75) antibody. **(A):** Labeling of flexor digitorum brevis (FDB) muscle‐derived NLSCs in culture with NGFR (p75) antibody. NLSCs are green owing to expression of GFP; NGFR antibody was detected using Alexa Fluor 647 secondary antibody (red color); all cells were counterstained with Hoechst nuclear stain (blue color). The merged picture shows the expression of NGFR, only on GFP^+^ NLSCs in the heterogeneous cell mixture derived from FDB muscles. This shows that NLSCs from nontransgenic animals can potentially be isolated using nerve growth factor cell surface receptor specific antibody (×20 magnification). **(B):** Representative histograms for FDB cultures before and after NGFR antibody‐based FACS. Histograms show purity of the obtained NGFR^+^ cell population. **(C):** Dot plot analysis showed a distinct GFP^+^/NGFR^+^ population. Abbreviations: DAPI, 4′,6‐diamidino‐2‐phenylindole; FACS, fluorescence‐activated cell sorting; GFP, green fluorescent protein; NGFR, nerve growth factor receptor.

NGFR expression was found to be unique to Nestin‐GFP^+^ NLSC population in heterogeneous cell cultures derived from FDB muscles of Nestin‐GFP^+^ transgenic mice in our previous studies 17. Confocal imaging of skeletal muscle cultures stained with anti‐NGFR antibody conjugated to Alexa Fluor 647 showed that NGFR expression was unique to GFP^+^ NLSCs and absent on non‐GFP^+^ cells (Fig. 2A). We therefore examined the potential to isolate NLSCs from heterogeneous skeletal muscle cell cultures by flow cytometry using only anti‐NGFR antibody. We successfully isolated a distinct population of NGFR^+^/GFP^+^ cells from skeletal muscle tissue cultures of Nestin‐GFP^+^ transgenic mice. Further FACS analysis indicated that the colocalization of NGFR antibody was exclusively to Nestin‐GFP^+^ cells (Fig. 2B). This result shows that a homogeneous population of NLSCs can be isolated by solely using NGFR‐based FACS without having to rely on the expression of a reporter gene, thereby highlighting clinical compatibility.

### Neural‐Like Stem Cells Show Tumor‐Tropic Behavior In Vitro

**Figure 3:**
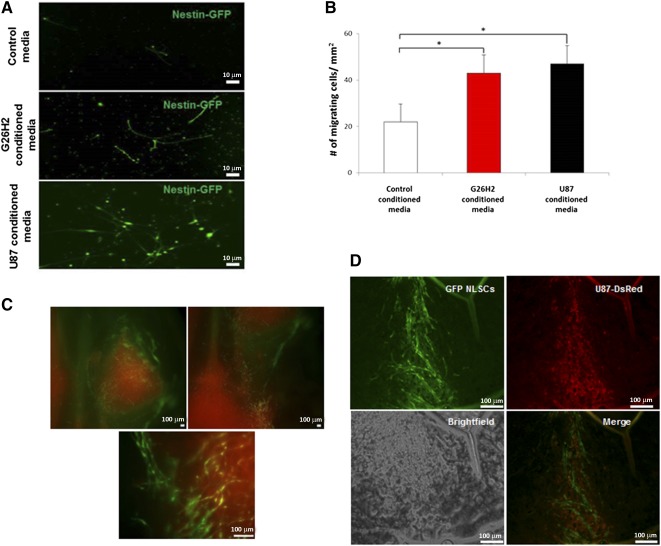
Neural‐like stem cells show tumor‐tropic behavior in vitro and in vivo. **(A):** Nestin‐GFP^+^ NLSCs in glioblastoma‐conditioned medium and control medium postmigration. NLSCs are green owing to expression of GFP under control of the Nestin promoter. Significant migration of GFP^+^ NLSCs toward G26H2‐conditioned media and U87‐conditioned media was observed when compared with their migration toward control media. Scale bars = 10 μm. **(B):** Graphical form of migration shown in panel A. Data are expressed as mean ± SEM (*n* = 3). The results indicated chemotactic behavior of Nestin‐GFP^+^ NLSCs toward cytokines secreted by G26H2 glioblastoma cell line. ∗, *p* < .05, Student's *t* test. **(C):** Fluorescence imaging of brain sections showing that intracranially injected GFP^+^ NLSCs (green) migrate toward orthotropic DsRed^+^ U87 tumors in vivo. Scale bars = 100 μm. **(D):** Extensive migration of GFP^+^ NLSC to site of DsRed^+^ U87 tumors was observed. Images show presence of migrated NLSCs within the tumor and its infiltrates. Scale bars = 100 μm. Abbreviations: GFP, green fluorescent protein; NLSC, neural‐like stem cells.

To initially demonstrate tumor tropism of our NLSCs, we performed an in vitro migration assay, as has been described previously 18. We incubated NLSCs with conditioned media from the G26H2 mouse glioblastoma cell line, the U87 human glioblastoma cell line, and control media. After 24 hours, we observed that the number of GFP^+^ NLSCs detected on the surface of wells containing glioma‐conditioned media were almost double when compared with control wells (Fig. 3A, 3B). This demonstrated the in vitro chemotactic affinity of NLSCs toward cytokines secreted by glioma cells. This tumor‐tropic nature of our NLSC population indicates the potential of NLSCs to be used as cellular delivery vehicles for anticancer therapeutics.

### Neural‐Like Stem Cells Show Tumor‐Tropic Behavior In Vivo

To demonstrate in vivo tumor tropism of our NLSCs, we examined the potential of intracranially injected peripherally derived NLSCs to migrate and infiltrate orthotropic GBM in vivo. NLSCs were implanted 2 mm adjacent to orthotropic DsRed^+^ U87 human glioblastoma tumors in nude mice. We observed extensive migration of our NLSCs toward the DsRed fluorescent tumors (Fig. 3C). We also observed NLSCs migrating precisely into the tumor projections (Fig. 3D). This confirmed the in vivo tumor tropism of NLSCs and their potential as therapeutic vehicles.

### NLSCs Do Not Stimulate Endothelial Tube Network Formation In Vitro

**Figure 4:**
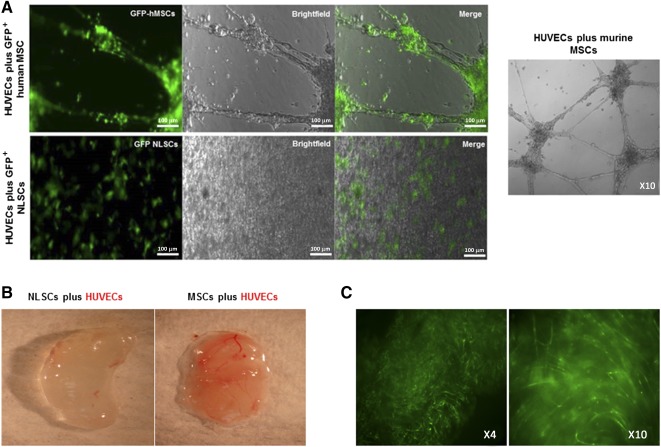
NLSCs do not stimulate endothelial tube network formation in vitro or in vivo. **(A):** Human MSCs (green) and murine MSCs cocultured with HUVECs for 14 days in Matrigel‐coated plates formed endothelial tubular structures. GFP^+^ NLSCs (green) cocultured with HUVECs under the same experimental conditions did not form endothelial tubular structures. The results show that HUVECs were stimulated by MSCs to form vascular structures in vitro, but in contrast, HUVECs were not stimulated to form such structures by NLSC. Scale bars = 100 μm. **(B):** Matrigel plugs extracted after 2 weeks from nude mice show contrasting vascularization. Matrigel plug containing NLSCs plus HUVECs shows no vascularization, whereas Matrigel plug containing MSCs plus HUVECs shows extensive vascularization. **(C):** GFP^+^ NLSCs inside the same Matrigel plug are observed under a fluorescence microscope (×4 and ×10 magnifications). Abbreviations: GFP, green fluorescent protein; h, human; HUVEC, human umbilical vein endothelial cell; MSC, mesenchymal stem cells; NLSC, neural‐like stem cells.

We investigated the potential of NLSCs to form endothelial tube networks in vitro when cocultured with HUVECs, as other stem cells, such as MSCs, have been shown to contribute to tumor angiogenesis. In contrast to what we observed with human and murine MSCs, we did not observe endothelial tip sprouting and stable vascular tube network in cultures of NLSCs and HUVECs (Fig. 4A). As was expected, control wells with HUVECs cultured alone formed unstable tube networks, which disappeared after a few days. These results show that our peripherally derived NLSCs do not stimulate HUVECs to form vessel‐like structures in vitro, in contrast to human and murine MSCs.

### NLSCs Do Not Contribute to Blood Vessel Formation In Vivo

In order to evaluate the angiogenic potential of NLSCs in vivo, we performed a standard Matrigel plug assay 14 by coincubating GFP^+^ MSCs or GFP^+^ NLSCs with HUVECs in Matrigel plugs, which were implanted subcutaneously in nude mice. Mice were monitored for 2 weeks to allow for the formation of vascular structures and later euthanized. We subsequently observed negligible blood vessel formation in Matrigel plugs containing HUVECs and NLSCs (Fig. 4B), despite the fact that fluorescence imaging showed abundant GFP^+^ NLSCs in the Matrigel plug (Fig. 4C). In contrast, we observed significant vessel formation in Matrigel plug‐ containing HUVECs and MSCs (Fig. 4B). These results demonstrate that our peripherally derived NLSC population does not stimulate HUVECs to form blood vessels in vivo, in contrast to MSCs.

### NLSCs Do Not Differentiate Into Tumor‐Associated Fibroblasts

**Figure 5:**
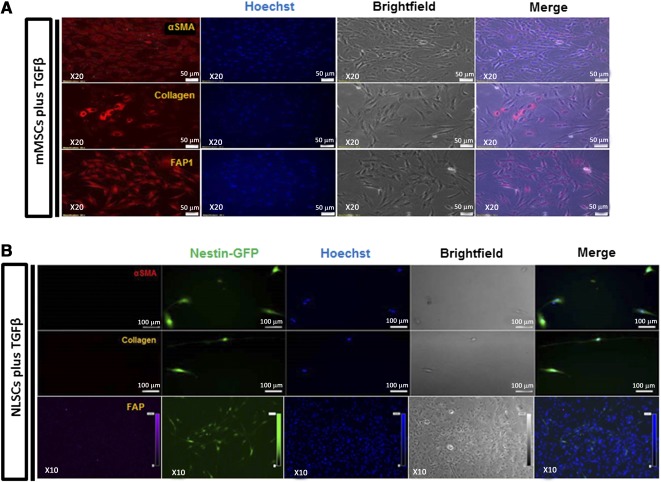
NLSCs do not differentiate into tumor‐associated fibroblasts. **(A):** Expression of αSMA, collagen type 1, and fibroblast‐associated protein cell surface markers by murine MSCs in vitro upon stimulation by TGF‐β‐supplemented media was observed under a fluorescence microscope using immunocytochemistry. Scale bars = 50 μm; ×20 magnification. **(B):** Absence of αSMA, collagen type 1, and fibroblast‐activated protein‐1 cell surface marker on GFP^+^ NLSCs after stimulation by TGF‐β‐supplemented media. Scale bars = 100 μm; ×10 magnification. Abbreviations: FAP, fibroblast‐associated protein; GFP, green fluorescent protein; m, murine, MSC, mesenchymal stem cells; NLSC, neural‐like stem cells; αSMA, α smooth muscle actin; TGF, transforming growth factor.

Murine NLSCs and MSCs were incubated with media containing TGF‐β1 to check for differentiation into TAFs. As was expected, we found enhanced expression of α‐SMA, collagen type 1 surface marker, and FAP‐1 in murine MSCs when exposed to TGF‐β1‐containing medium (Fig. 5A). In contrast, we did not observe the expression of FAP‐1, α‐SMA, or collagen type 1 cell surface markers on our NLSC population (Fig. 5B). These results indicate that our peripherally derived NLSCs do not transform into TAFs, which have been shown to contribute to tumor aggressiveness 19.

### NLSCs Can Be Genetically Engineered to Express a Prototype Therapeutic

**Figure 6:**
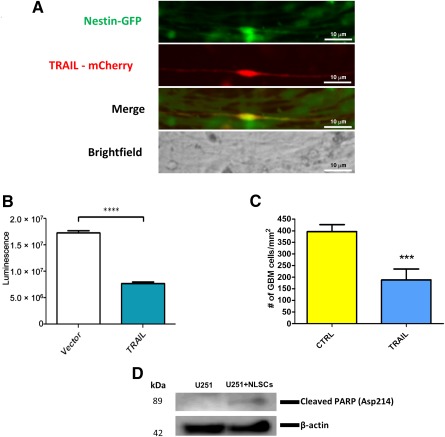
NLSCs can be genetically engineered for therapeutic purposes. **(A):** TRAIL‐mCherry expression by engineered NLSC. NLSCs were transfected with a plasmid encoding TRAIL‐mCherry under the control of the cytomegalovirus promoter. Scale bars = 10 μm. **(B):** Cytotoxic effect of TRAIL‐expressing NLSCs against U251 cell line measured by the luminescence signal from culture plates. ****, Significant reduction (*p* < .001) in the luminescence signal from U251 cultures containing engineered NLSCs was observed when compared with control cultures. **(C):** Cytotoxic effect of TRAIL‐expressing NLSCs against U251 cell line measured by counting the number of viable U251 cells. ***, Significant decrease (*p* < .001) in the number of viable U251 cells was observed in cultures containing engineered NLSCs when compared with control cultures. **(D):** Western blotting showed the presence of cleaved PARP, which is an indication of TRAIL‐induced apoptosis in U251 cultures containing engineered NLSCs. Cleaved PARP was not detected in control cultures. Abbreviations: CTRL, control; GBM, glioblastoma; GFP, green fluorescent protein; NLSC, neural‐like stem cells; PARP, poly ADP ribose polymerase; TRAIL, tumor necrosis factor‐(TNF)‐α‐related apoptosis‐inducing ligand.

NLSCs were infected with a lentivirus containing the TRAIL transgene, coexpressed with a mCherry reporter gene. We demonstrate expression of transgene via mCherry fluorescence (Fig. 6A), confirming our ability to genetically engineer NLSCs for therapeutic purposes. To evaluate the antitumor activity of NLSCs expressing mCherry‐TRAIL, we cocultured TRAIL‐engineered or control NLSCs together with luciferase‐expressing GBM cells. After 24 hours, we observed a significant decrease in bioluminescence signal and GBM cell number in wells containing cocultures of GBM cells and TRAIL‐expressing NLSCs, but not in controls (Fig. 6B, 6C). Furthermore, we found significantly increased poly ADP ribose polymerase cleavage in cells cocultured with TRAIL‐expressing NLSCs (Fig. 6D), an indication of TRAIL‐induced apoptosis 20. These results show the potential ability of NLSCs to be genetically engineered and used as therapeutic carriers against glioblastomas.

## Discussion

Tumor microenvironments are often characterized by extracellular signaling networks, which are involved in the recruitment of various stem cells 21. Many systemically administered chemotherapeutics have been disappointing against GBM, because of ineffective concentrations reaching infiltrating tumor cells. This problem has been attributed to the vascular impediments of the blood‐brain barrier and the complex vascular networks of tumor microenvironments. Therefore, many groups have investigated engineered stem cells to deliver drugs into the tumor microenvironment as a complementary strategy 22.

**Figure 7:**
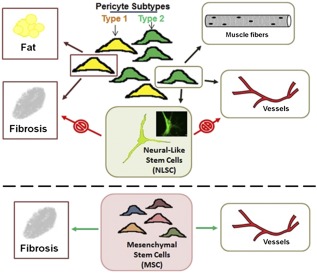
Pericyte heterogeneity and the origin of NLSCs. We discovered that there are 2 subsets of pericytes that have distinct differentiation potentials. Type 1 pericytes (yellow: Nestin^−^/NG2^+^) appear to differentiate into fat and fibroblasts. In contrast, type 2 pericytes (green: Nestin^+^/NG2^+^) differentiate into muscle fibers, NLSCs, and support angiogenesis. We found that these NLSCs do not differentiate into fibroblasts (Fig. 5). In contrast, heterogeneous populations of MSCs can differentiate into fibroblasts, including tumor‐associated fibroblasts, which can potentially aid tumor cell growth and resistance. Additionally, NLSCs did not contribute to angiogenesis, in contrast to MSCs, as demonstrated in an in vivo plug assay (Fig. 4). Abbreviations: MSCs, mesenchymal stem cells; NLSCs, neural‐like stem cells.

We recently discovered a new pure population of neural‐like stem cells derived from muscle pericytes. Pericytes are multipotent cells found around vessels of all tissue types and are generally categorized within the classification of mesenchymal stem cells 23. Pericytes, as a heterogeneous group, have been shown to have the potential to differentiate into various lineages, including myogenic, adipogenic, osteogenic, chondrogenic, and endothelial 18, 24, 25, 26, 27. We recently demonstrated for the first time that there are two distinct pericyte subtypes by using transgenic mice that express GFP and red fluorescent protein under the Nestin and NG2 promoters. Using our unique double transgenic mice, we found that some pericytes express only NG2 (type 1 pericytes), whereas other pericytes express both NG2 and Nestin (type 2 pericytes) 15, 17, 28, 29. Of great significance, we discovered that these two pericyte subtypes differentiate into distinct lineages (Fig. 7) 23, 30, 31. Importantly, we found that type 2 pericytes can be differentiated into a “neural‐like” stem cell (NLSC) similar to NG2 glia after a few days in culture under specialized conditions and give rise to cells that (a) express a number of specific neural NG2‐glia markers; (b) demonstrate replicative capacity; (c) have the ability to form neurospheres; and (d) demonstrate a functional response to a neurotransmitter (Fig. 7) 15, 17, 32, 33, 34.

Furthermore, we and others established that these novel NLSCs are also expressed in other species, including rats, canines, and nonhuman primates 15, 35, 36, 37, 38, 39. We successfully obtained these NLSCs from additional peripheral tissues and quantified NLSCs per milligram of tissue 17. We were able to isolate the largest number of NLSCs from muscle (30,000 per milligram), followed by bone marrow (∼2,000 per milligram), with the least amount isolated from adipose tissue (113 per milligram) 17. These NLSCs exhibit increasing glutamate‐evoked membrane currents as time passed in differentiation media, indicating progressive differentiation and expression of ion channels in culture 15. Given these findings, we hypothesized that NLSCs represented a more distinct and differentiated class of potential progenitor cells that would home to tumor cells, similar to NSCs, but would not transform into TAFs or contribute to tumor angiogenesis because of their lineage and more differentiated state in comparison with MSCs (Fig. 7).

We examined the potential for using our newly isolated peripherally derived NLSCs as drug‐ delivery vehicles to GBM. After initial identification and isolation of NLSCs using Nestin‐GFP transgenic mice, we also for the first time successfully isolated NLSCs from skeletal muscle cultures using nerve growth factor surface receptor via FACS, thereby highlighting their clinical relevance. We also demonstrated in vitro and in vivo that NLSCs exhibit chemotaxis toward cytokines secreted by gliomas, similar to what is seen with other stem cells. Recurrence of disease posttreatment due to untreated infiltrating GBM cells is a major problem when treating brain tumor patients 40. We found that NLSCs migrated to infiltrating tumor projections, showing their potential to be considered as therapeutic delivery vehicles.

In regard to angiogenesis, although we have demonstrated that type 2 pericytes have the potential to contribute to vasculogenesis both in tumors and in in vivo vascular plug assays 16, 23, 41, we found that once the type 2 pericytes differentiate into NLSCs, they no longer had the potential to participate in vasculogenesis (Fig. 7), as results from our in vitro and in vivo angiogenesis experiments showed that NLSCs do not activate HUVECs to form vascular structures (Figs. 4, 5). A lack of paracrine secretion of angiogenesis‐inducing cytokines by NLSCs could possibly explain this observation 42.

Despite the ease of availability and culture in vitro, commonly used peripherally derived stem cells, such as MSCs were observed to enhance tumor progression, angiogenesis, and metastasis by transforming into TAFs over time by using the CXCR12/CXCR4 axis and vascular endothelial growth factor signals within the tumor microenvironment 43. Because NLSCs are differentiated from type 2 pericytes that did not transform or contribute to injury‐associated fibroblasts/fibrosis (Fig. 7), we hypothesized that NLSCs do not have fibrogenic potential, in contrast to what has been reported with MSCs. We found that upon being exposed in vitro to fibrogenic media‐containing cytokines involved in transforming stromal cells into TAFs 44, NLSCs did not show the expression of α‐SMA, FAP‐1, and collagen type 1 cell surface markers, which are markers of TAFs 19. The lack of transformation into TAFs presented in our results indicates that NLSCs may not contribute toward progression and epithelial‐mesenchymal transition of tumors upon entering cell signaling networks in the tumor microenvironment, which may indicate a significant advantage over other stem cells commonly used for delivering therapeutics to tumor sites. This is currently an active area of our exploration.

Some peripherally derived stem cells have been shown to form teratomas or teratocarcinomas upon implantation in vivo 45. When injected in vivo for 6 months (subcutaneously or orthotopically), our NLSCs did not form any such teratomas or teratocarcinomas (data not shown), highlighting their potential safety and feasibility for clinical usage. Furthermore, our proof‐of‐concept in vitro TRAIL experiment demonstrated the ability of our NLSC population to be genetically manipulated for therapeutic purposes. The successful secretion of TRAIL, a prototype anticancer agent, and subsequent cytotoxicity of adjacent glioblastoma cell line emphasize the therapeutic potential of NLSCs.

## Conclusion

We demonstrated tumor tropism of newly isolated peripherally derived NLSCs that do not form tumors or contribute to angiogenesis in vivo. Furthermore, our NLSCs did not differentiate into TAFs, unlike MSCs. We also showed the potential of NLSCs to be genetically engineered for therapeutic purposes and are in the process of engineering NLSCs to secrete other novel GBM‐targeted therapeutics.

## Author Contributions

A.B.: conception and design, experiment design, collection and/or assembly of data, data analysis and interpretation, final approval of manuscript; A.S.: experiment design, collection and/or assembly of data, data analysis and interpretation, manuscript writing, final approval of manuscript; D.Z., G.Z., I.B., V.T.N., and M.L.M.: collection and/or assembly of data, final approval of manuscript; K.K.S.S.: experiment design, critical review of the manuscript, final approval of manuscript; F.C.M.: experiment design, final approval of manuscript; O.D.: conception and design, critical review of the manuscript, final approval of manuscript; A.M.: conception and design, experiment design, data analysis and interpretation, manuscript writing, critical review of the manuscript, final approval of manuscript.

## Disclosure of Potential Conflicts of Interest

The authors indicated no potential conflicts of interest.
